# Mutations in *B4GALNT1* (GM2 synthase) underlie a new disorder of ganglioside biosynthesis

**DOI:** 10.1093/brain/awt270

**Published:** 2013-10-06

**Authors:** Gaurav V. Harlalka, Anna Lehman, Barry Chioza, Emma L. Baple, Reza Maroofian, Harold Cross, Ajith Sreekantan-Nair, David A. Priestman, Saeed Al-Turki, Meriel E. McEntagart, Christos Proukakis, Louise Royle, Radoslaw P. Kozak, Laila Bastaki, Michael Patton, Karin Wagner, Roselyn Coblentz, Joy Price, Michelle Mezei, Kamilla Schlade-Bartusiak, Frances M. Platt, Matthew E. Hurles, Andrew H. Crosby

**Affiliations:** 1 Institute of Biomedical and Clinical Science, University of Exeter Medical School, St. Luke’s Campus, Heavitree Road, EX1 2LU, Exeter, Devon, UK; 2 Child and Family Research Institute, The University of British Columbia, 950 West 28th Ave, Vancouver, BC, V5Z 4H4, Canada; 3 Department of Ophthalmology and Vision Science, University of Arizona School of Medicine, Tucson, AZ 85711, Arizona, USA; 4 Department of Pharmacology, University of Oxford, Oxford, OX1 3QT, UK; 5 Wellcome Trust Sanger Institute, Wellcome Trust Genome Campus, Hinxton, Cambridge, CB10 1SA, UK; 6 Medical Genetics Unit, Floor 0, Jenner Wing, St. George’s University of London, Cranmer Terrace, London SW17 0RE, UK; 7 Department of Clinical Neuroscience, Institute of Neurology, University College London, Queen Square, London WC1N 3BG, UK; 8 Ludger Ltd., Culham Science Centre, Oxfordshire, OX14 3EB, UK; 9 Kuwait Medical Genetics Centre, Ghanima Alghanim Building, Maternity Hospital, Sulaibikhat, Postal Code: 80901, Kuwait; 10 Windows of Hope Genetic Information Centre, Holmes County, Walnut Creek, OH 44065 Ohio, USA; 11 Division of Neurology, The University of British Columbia, UBC Hospital S192 – 2211 Wesbrook Mall, V6T 2B5, Canada

**Keywords:** ganglioside biosynthesis, *B4GALNT1*, Amish, SPG26, hereditary spastic paraplegia

## Abstract

Glycosphingolipids are ubiquitous constituents of eukaryotic plasma membranes, and their sialylated derivatives, gangliosides, are the major class of glycoconjugates expressed by neurons. Deficiencies in their catabolic pathways give rise to a large and well-studied group of inherited disorders, the lysosomal storage diseases. Although many glycosphingolipid catabolic defects have been defined, only one proven inherited disease arising from a defect in ganglioside biosynthesis is known. This disease, because of defects in the first step of ganglioside biosynthesis (GM3 synthase), results in a severe epileptic disorder found at high frequency amongst the Old Order Amish. Here we investigated an unusual neurodegenerative phenotype, most commonly classified as a complex form of hereditary spastic paraplegia, present in families from Kuwait, Italy and the Old Order Amish. Our genetic studies identified mutations in *B4GALNT1* (GM2 synthase), encoding the enzyme that catalyzes the second step in complex ganglioside biosynthesis, as the cause of this neurodegenerative phenotype. Biochemical profiling of glycosphingolipid biosynthesis confirmed a lack of GM2 in affected subjects in association with a predictable increase in levels of its precursor, GM3, a finding that will greatly facilitate diagnosis of this condition. With the description of two neurological human diseases involving defects in two sequentially acting enzymes in ganglioside biosynthesis, there is the real possibility that a previously unidentified family of ganglioside deficiency diseases exist. The study of patients and animal models of these disorders will pave the way for a greater understanding of the role gangliosides play in neuronal structure and function and provide insights into the development of effective treatment therapies.

## Introduction

Glycosphingolipids (GSLs) are ubiquitous constituents of eukaryotic plasma membranes, and their sialylated derivatives, gangliosides, are the major class of glycoconjugates expressed by neurons (Schnar, 2005). Deficiencies in their catabolic pathways give rise to excessive intralysosomal accumulation of these lipids associated with a large and well-studied group of inherited disorders, the lysosomal storage diseases (Platt and Walkley, 2004; Ballabio and Gieselmann, 2009). These disorders typically involve progressive neurodegeneration and, for the majority of these diseases, no effective treatment is available (Platt and Lachmann, 2009). Although many GSL catabolic defects have been defined to date (Ginzburg *et al.*, 2004), very few are known to result from a defect in GSL biosynthesis and previous reports of potential ganglioside biosynthesis disorders based on biochemical evidence have not been proven genetically (Fishman *et al.*, 1975). Proven disorders include a defect in serine palmitoyltransferase long chain subunit 1 (SPTLC1), which catalyzes the first step in sphingolipid biosynthesis leading to downstream ceramide and sphingolipids biosynthesis, which is associated with hereditary sensory and autonomic neuropathy (HSAN1) (Bejaoui *et al.*, 2001). However, these patients retain the ability to synthesize GSLs by using L-alanine as the precursor instead of serine. This results in the production of an atypical class of deoxy-sphingolipids. A more selective defect has also been described, termed ‘GM3 synthase deficiency’, which was previously the only proven defect in ganglioside biosynthesis. This novel human disease is a severe epileptic disorder identified amongst families of the Old Order Amish community (Simpson *et al.*, 2004). Its existence led us to consider that other human diseases, not yet identified, may exist that result from defects in enzymes that function at different steps in the ganglioside biosynthetic pathway (Lannert *et al.*, 1998; Daniotti and Iglesias-Bartolome, 2011). As gangliosides are conserved lipids, expressed at high levels in the nervous system of mammals, we hypothesized that any such conditions would likely display neurological (particularly neurodegenerative) phenotypes.

**Figure 1:**
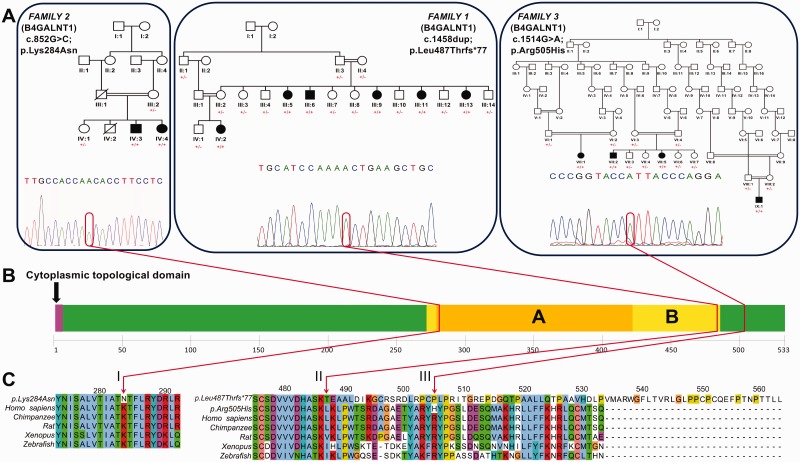
(**A**) Pedigrees of Families 1 (Kuwaiti), 2 (Italian) and 3 (Old Order Amish). Segregation of c.1458dup (exon 10; p.Leu487Thrfs*77), c.852G>C (exon 7; p.Lys284Asn) and c.1514G>A (exon 10; p.Arg505His) variants throughout each family with mutant allele indicated by ‘+’ and wild-type allele indicated by ‘−’, as well as the sequence chromatogram for each variant. (**B**) The position of each sequence variant with regard to domain architecture of the protein where ‘A’ refers to ‘glycosyl transferase, Family 2 domain’, and ‘B’ indicates ‘nucleotide-diphospho-sugar transferases superfamily domain’. (**C**) Species conservation alignments for the regions affected by each variant.

In the current study, we investigated the genetic and molecular basis of an unusual neurodegenerative phenotype, best described as a complex form of hereditary spastic paraplegia. The hereditary spastic paraplegias are among the most genetically diverse of all inherited Mendelian disorders, with >50 distinct loci and >35 genes identified to date. They comprise a large group of neurodegenerative diseases characterized clinically by the presence of lower limb spasticity, and pathologically by the retrograde degeneration of the longest nerve fibres in the corticospinal tracts and posterior columns. The genes known to underlie hereditary spastic paraplegia seem to encode molecules with a diverse range of cellular functions, implicating numerous defective biological processes in the pathogenesis of this disease (Blackstone *et al.*, 2011). We previously mapped a gene for a complex autosomal recessive form of hereditary spastic paraplegia (SPG26) in a large Kuwaiti family (Family 1, Fig. 1A) to a ∼34 Mb region of chromosome 12q (Wilkinson *et al.*, 2005). In the current study, we determined that mutations in *B4GALNT1*, encoding GM2 synthase, are responsible for this condition. We also identified mutations in this gene in an Italian-Canadian family as well as families from the Old Order Amish community, in which the only other known disorder relating to defective ganglioside biosynthesis has also been described.

## Materials and methods

### Subjects

Blood or buccal samples were obtained from affected children, parents and unaffected family members with informed consent. DNA and RNA were extracted by standard procedures. A single 0.4-mm diameter skin biopsy was taken from individuals from Family 2 IV:3, IV:4 (affected) and IV:1 (unaffected), and family 3 VII:2 and VII:5 (affected). All samples were obtained with approved informed consent. All affected individuals underwent a detailed physical examination.

### Exome and targeted regional sequencing

Exome sequencing was undertaken in a single affected individual from each family, using the SureSelect All Exons 50 Mb Target Enrichment System (Agilent) and sequencing on a HiSeq (Illumina) with 76 bp paired end reads. Single nucleotide variants were called using two programs (GATK, SamTools) while insertion-deletions (indels) were called using (GATK and Dindel). All variants were annotated using dbSNP (132) and the 1000 Genomes pilot study. Single nucleotide polymorphism effect predictor (VEP) was used for predicting the variant consequences on the protein structure based on Ensembl (version 61). Both single nucleotide variants and indels called by the three programs were then merged into a single file for downstream analysis. Variants were filtered out if the read depth was <4× or >1200×, if the consensus quality was <20 or if the single nucleotide polymorphism quality was <25.

### Genotyping and linkage analysis

Single nucleotide polymorphism microarray genotyping was performed using Illumina Human CytoSNP-12v2.1 (Family 3) and Affymetrix 6.0 single nucleotide polymorphism (Family 2) arrays. Marker saturation was carried out using existing and newly generated microsatellite markers (primer sequences available on request). Alleles were size-fractionated using 8% polyacrylamide gels and DNA was visualized by silver staining.

### Mutation analysis

Unique sequence intronic primers were designed to amplify all potentially pathogenic variants identified within the critical region. Purified PCR amplification products were sequenced using dye-terminator chemistry and electrophoresed on an ABI 3130XLA capillary sequencer (Applied Biosystems). Mutation analysis was carried out using Mutation Surveyor software v3.20 (SoftGenetics). Both strands of each product were sequenced.

### Fibroblast culture

Human forearm fibroblasts were cultured in Dulbecco’s modified Eagle medium with 10% foetal bovine serum and antibiotics, after standard protocols in 75 cm flasks. Cells were dissociated from flasks for analysis by means of 5 min incubation with 0.05% trypsin with 0.02% EDTA at 37°C. Fibroblast pellets were taken up in 0.5 ml Milli-Q water and freeze-thawed three times. GSL extraction, isolation, 2AA-labelling and HPLC were performed as previously described (Neville *et al.*, 2004).

### Exoglycosidase digests

Labelled glycans released from fibroblast GSLs were digested for 18 h at 37°C in a total volume of 20 µl with sialidase A, α-galactosidase, β-galactosidase and β-hexosaminidase (Prozyme/Glyko) according to manufacturer’s instructions. Following digestion, labelled glycans were separated from enzyme protein using Corning® Costar® Spin-X® centrifuge tube filters before HPLC, as above.

### MALDI mass spectrometry

Released glycans were permethylated (Ciucanu and Costello, 2003) then analysed on a Shimatzu Resonance MALDI instrument using 2,5-dihydroxybenzoic acid (DHB) matrix per-O-methylation of neutral carbohydrates directly from aqueous samples for gas chromatography and mass spectrometry analysis (Ciucanu and Caprita, 2007). Samples were analysed with two different mass range settings: 350–850 and 850–2000 Da.

## Results

### Genetic studies

We previously detailed the clinical features of a large Kuwaiti family with a complex neurodegenerative phenotype (Wilkinson *et al.*, 2005) and a clinical description of the additional Italian-Canadian and Old Order Amish families is supplied in the Supplementary material. The most prominent clinical phenotype common to these families is progressive weakness and spasticity, affecting the lower extremities, plus or minus the upper extremities. All affected individuals also manifest apparently non-progressive cognitive impairment. Additional features included cerebellar signs, sensory polyneuropathy, pes cavus, stereotypies, emotional lability and psychiatric illness. Seizure activity was reported in two of four Amish individuals. Where neuroimaging was undertaken no abnormalities were reported. Therefore, a clinical diagnosis of complicated hereditary spastic paraplegia had been assigned to these families, although there are additional features present in some affected individuals that provide evidence of a broader phenotype associated with GM2 synthase deficiency.

To robustly position the gene responsible for this condition, we first refined the extent of the disease locus previously defined in the Kuwaiti family using microsatellite markers to chr12:52 012 214–65 342 437, a ∼13.33 Mb region containing ∼317 genes (data not shown). In order to identify the causative mutation we designed a custom 1× tiling bait array to capture a targeted region encompassing this region. The target size was ∼6.25 Mb after repeat masking, generating 6.2 Gb of data with a mean depth of 570. After filtering and exclusion of novel sequence variants found to be present in homozygous state in Kuwaiti controls, only a single likely deleterious variant remained as potentially causative. This was a frameshift insertion in the last exon of the beta-1,4-*N*-acetyl-galactosaminyl transferase 1 (*B4GALNT1*) gene (Fig. 1; chr12:g.58020671dup; NM_001478.3: c.1458dup, p.Leu487Thrfs*77), which may therefore be likely to encode a transcript that escapes nonsense-mediated messenger RNA decay surveillance mechanisms and produces a polypeptide with an altered and elongated C-terminus (Fig. 1)*.* Dideoxy sequence analysis revealed that the variant cosegregated with the disease phenotype, with affected individuals being homozygous for the variant, and parents and all unaffected offspring being heterozygous carriers (Fig. 1). In parallel with this targeted regional sequencing approach, whole exome sequencing was undertaken using DNA from a single affected individual from two additional families with a clinically similar presentation to the Kuwaiti family. The first of these families, who originate from a small village in the Frosinone region of central Italy, have two affected offspring from a consanguineous union (Family 2, Fig. 1). After filtering of variants only two novel likely deleterious sequence variants were found to be present in genes located in shared homozygous regions between the two affected cases detected by a complementary Affymetrix 6.0 single nucleotide polymorphism microarray scan (data not shown), both of which cosegregated with the disease phenotype as determined by dideoxy sequence analysis. One was located in *OR51S1* (NM_001004758.1: c.821C>T; p.Pro274Leu), an olfactory receptor gene considered an unlikely candidate to cause this condition. The second variant affects a stringently conserved residue in *B4GALNT1* (Fig. 1; chr12:g.58022646G>C; NM_001478.3:c.852G>C; p.Lys284Asn) and is predicted to be damaging by PolyPhen-2, SIFT and PROVEAN. The second DNA sample subject to exome sequencing was from a single affected individual from an extended Amish pedigree (Family 3, Fig. 1). This identified another homozygous likely deleterious sequence alteration in *B4GALNT1* (Fig. 1; chr12:g.58020615C>T; NM_001478.3: c.1514G>A; p.Arg505His), located in ∼15 Mb region of homozygosity delimited by markers rs7139287 and rs2717445 (data not shown) shared by four affected individuals in three Amish nuclear families. This variant cosegregates with the disease phenotype as determined by dideoxy sequence analysis and is predicted deleterious by PolyPhen-2, SIFT and PROVEAN. It was found to be present in a heterozygous state in two out of 300 Amish control subjects, which is not unexpected of a founder mutation present in the community. The Amish variant is not listed in the 1000 Genomes genomic database (comprising 2184 chromosomes) and only a single heterozygous carrier of European origin was identified out of 13 006 chromosomes analysed in the National Heart, Lung and Blood Institute Exome Sequencing Project database (ESP6500SI release). Neither the *B4GALNT1* c.1458dup frameshift nor c.852G>C variants are present in publicly accessible genomic databases, or in 100 ethnically matched control chromosomes.

### Biochemical studies

**Figure 2:**
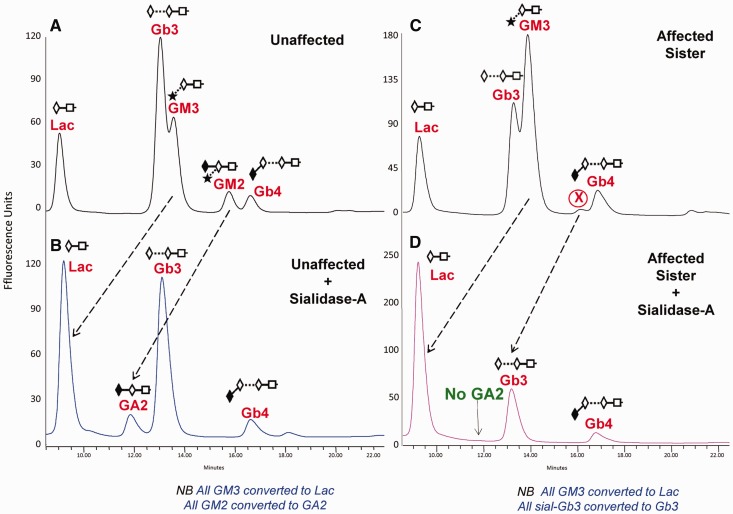
Representative HPLC profiles for 2-AA labelled glycans released from GSLs isolated from cultured human skin fibroblasts. Unaffected patient (control; **A** and **B**). Affected sister/sibling (**C** and **D**) in the Italian family. **B** and **D** show the peak shifts, indicated with dashed arrows, following digestion of the labelled glycans with Sialidase A. Peak ‘X’ (in **C**) is a previously unreported GSL, sialylated Gb3.

As the *B4GALNT1* variants identified were predicted to abolish GM2 synthase activity of the encoded polypeptide, we investigated the biochemical effect of the mutation in cultured skin fibroblasts from an affected brother and sister from the Italian family, as these were available for investigation. GM2 ganglioside is a sialylated GSL that is synthesized in the Golgi apparatus as part of a complex biosynthetic pathway (Supplementary Fig. 1). GM2 synthase is a GalNAc transferase responsible for synthesizing GM2, GA2 and GD2 (Pohlentz *et al.*, 1988). If GM2 synthase were deficient in the affected individuals, no GM2, GA2, GD2 or downstream derivatives would be synthesized (blue box, Supplementary Fig. 1). To test this hypothesis, fibroblasts from both affected individuals were analysed by generating fluorescently-labelled GSL-derived oligosaccharides and performing HPLC (Fig. 2) (Neville *et al.*, 2004). We confirmed the glycan structures of the GSL-derived oligosaccharides in healthy individuals by comparing them with commercially available authentic standards, by performing exoglycosidase digestion and by confirming structures by mass spectrometry (Supplementary Figs 2 and 3 and Supplementary Table 1). A representative profile of fibroblast GSLs from an unaffected individual (Fig. 2A) showed that the major GSL species detectable were LacCer, Gb3, GM3, GM2 and Gb4. HPLC profiles from the two affected patients’ fibroblasts (Family 2 Individuals IV:3 and IV:4) were identical to each other, but differed in several ways from unaffected fibroblasts. Firstly, there was an increase in the level of GM3 relative to Gb3 (Fig. 2C). Secondly, there was a large reduction in the GM2 peak (authenticated by mass spectrometry, Supplementary Fig. 2, in the unaffected patient fibroblasts), leaving only a minor peak ‘X’, in affected patient cells. To determine the structure of peak ‘X’, we performed sialidase-A digests on both affected and unaffected fibroblast samples. As expected, the GM3 glycan was digested to Lac, confirming this structure in both samples. In the unaffected fibroblasts the GM2 peak was converted to asialo GM2 (GA2) confirming it as GM2 (Fig. 2B). Peak ‘X’ in the affected patients (Fig. 2C) was also sensitive to sialidase digestion (Fig. 2D). However, in contrast with the unaffected sample (Fig. 2B), it did not lead to the production of GA2 and instead, was converted into either Gb3 or LacCer, ruling out the presence of GM2 in the affected patient fibroblasts (Fig. 2D). To establish more specific structural information, preparative HPLC was performed collecting individual peaks (Fig. 2C). When peak ‘X’ was digested with sialidase-A, the peak shifted to a retention time consistent with Gb3, revealing the structure to be a previously unreported novel GSL namely sialylated Gb3. To explain this, we hypothesize that in the absence of functional GM2 synthase in the patient cells there will be a lack of downstream GSLs as substrates for sialyltransferase, ST3GALII, and this may be the cause of the ectopic sialylation of Gb3. Absolute quantities of each individual GSL peak were determined and are summarized (Supplementary Table 2). The two affected siblings showed remarkably similar absolute amounts of GSLs whereas the control sample had ∼25% less, in total, suggesting that the patients may have not only altered composition but also increased GSL levels. Analysis of more samples will be required to verify this observation. In addition to the sialidase digests, further digests with α-galactosidase, β-galactosidase and β-hexosaminidase confirmed the presence of Gb3, LacCer and Gb4 glycan structures in both samples (data not shown). Similar biochemical findings were seen when analysing fibroblasts from the Amish pedigree (Individual IX:1; Supplementary Fig. 2).

## Discussion

In the CNS of mammals, ganglioside structures and their expression levels are highly conserved. Four gangliosides predominate, namely GM1, GD1a, GD1b and GT1b (Schnaar, 2010). Insights into the functions of gangliosides have come in part from studying engineered mice deficient in key glycosyltransferases required for ganglioside biosynthesis (Schnaar, 2010). For example, when B4GALNT (GM2 synthase, the enzyme deficient in the patients in the current study) is deficient in the mouse, none of the major brain gangliosides are synthesized. Instead, the simple gangliosides GM3 and GD3 are present at higher than normal levels, as they are synthesized before the biosynthetic block (Takamiya *et al.*, 1996; Kawai *et al.*, 2000). Indeed, total GSL levels are comparable in both wild-type and engineered mice, with increased levels of ‘pre-block’ simple GSLs compensating for the lack of complex ganglioside biosynthesis (Sun *et al.*, 2004) illustrating tight control of total GSL levels. As GD3 does not bind to myelin-associated glycoprotein (MAG), a key mediator of myelin stability, and GM3 binds poorly to myelin-associated glycoprotein (Collins *et al.*, 1997), these mice have defective axon:myelin stability leading to progressive neuropathy, similar to that seen in MAG-deficient mice (Sun *et al.*, 2004). Wallerian axonal degeneration occurs in B4GALNT1 mice leading to impaired motor coordination and diminished hind-limb reflexes (Sheikh *et al.*, 1999). In addition, the mice displayed abnormal structures at the nodes of Ranvier, leading to electrophysiological defects characterized by reduced conduction velocities in motor nerves (Susuki *et al.*, 2007). It remains to be determined the extent to which the clinical features of the patients in this study and the engineered GM2 synthase mouse converge, but loss of complex gangliosides could potentially disrupt axon:myelin stability in both mouse and man.

To date, only one other human inherited disease resulting from a proven ganglioside biosynthetic defect has been described, namely GM3 synthase deficiency present amongst the Old Order Amish (Simpson *et al.*, 2004). This presents clinically as a severe neurological syndrome, which in most patients involves intractable seizures, but not in the mouse model (Yamashita *et al.*, 2003), likely because of compensation through o-series GSL biosynthesis (Supplementary Fig. 1). Here we describe a second human neurological disease involving a defect in the adjacent enzyme acting in the ganglioside biosynthetic pathway in families of different ethnic backgrounds, including the Amish in which both known ganglioside disorders are present. All families show a phenotype consisting mainly of progressive spastic paraparesis, together with variable learning difficulties, although this doesn’t appear to be progressive. Clinical examination and nerve conduction suggest a mild peripheral neuropathy in some of the cases. The siblings in Family 2 share several unusual traits in addition to paraplegia and intellectual disability: congenital cervical spinal narrowing, dysmorphological features (prognathism, wide mouth, deep-set eyes), and tumours (ovarian teratoma and colonic adenocarcinoma). These features may be merely coincidental, may relate to another genetic disorder, or more likely be connected to this disorder of ganglioside biosynthesis. The earlier report of a patient with the biochemical hallmarks of GM2 synthase deficiency at post-mortem (Fishman *et al.*, 1975) remains genetically unproven. Whether that report describes a more severe phenotype arising from GM2 synthase deficiency remains unclear, although this may be unlikely as the mutations described in the current study and by Boukhris *et al.* (2013) are all predicted to result in complete loss of enzyme activity, which has been confirmed biochemically in Families 2 and 3 in the current study. Consequently it therefore remains impossible to determine the relationship between the cases of proven GM2 synthase deficiency in the current study, and the historic case report. However, the biochemical assay we employed may be of use diagnostically in the future in individuals with clinical features reminiscent of the complex form of hereditary spastic paraplegia described here.

With the description now of two neurological human diseases involving defects in two sequentially acting enzymes involved in the ganglioside biosynthetic pathway, there is the real possibility that a previously unidentified family of ganglioside deficiency diseases exists. The study of patients and animal models of these disorders will pave the way for greater understanding of the role gangliosides play in neuronal structure and function and provide insights into the future development of effective therapies to treat patients afflicted with these devastating disorders.

## Web resources

The URLs for data presented herein are as follows:

GATK; http://www.broadinstitute.org/gatk/about/citing-gatk

VEP; http://www.ensembl.org/info/docs/variation/vep/index.html

SAMTOOLS; http://samtools.sourceforge.net

NHLBI Exome Sequencing Project (ESP); http://evs.gs.washington.edu/EVS/

Online Mendelian Inheritance in Man; http://www.ncbi.nlm.nih.gov/Omim

Pubmed; http://www.ncbi.nlm.nih.gov/pubmed/

Gene; http://www.ncbi.nlm.nih.gov/gene

ClustalW2; http://www.ebi.ac.uk/Tools/msa/clustalw2/

PolyPhen-2; http://genetics.bwh.harvard.edu/pph2/

SIFT; http://sift.jcvi.org/

PROVEAN; http://provean.jcvi.org/seq_submit.php

GeneCards; http://www.genecards.org/

The 1000 Genomes Browser; http://browser.1000genomes.org/index.html

The Ensembl Project; http://www.ensembl.org/index.html

The National Center for Biotechnology; http://www.ncbi.nlm.nih.gov/

UCSC Human Genome Database; http://www.genome.ucsc.edu

## Accession Numbers

The databank accession number for *B4GALNT1* reported in this paper is NM_001478.3.

## Supplementary Material

Supplementary Data

## Abbreviation

GSL: glycosphingolipid
