# Supplementary material for: Mutations in B4GALNT1 (GM2 synthase) underlie a new disorder of ganglioside biosynthesis
Source: Brain. 2013 Oct 6;136(12):3618–24. doi: 10.1093/brain/awt270 (PMC3859217; doi:10.1093/brain/awt270)
Supplement: Supplementary Data [file supp_136_12_3618__index.html]

Mutations in B4GALNT1 (GM2 synthase) underlie a new disorder of ganglioside biosynthesis — Supplementary Data 

# Mutations in *B4GALNT1* (GM2 synthase) underlie a new disorder of ganglioside biosynthesis

## Supplementary Data

files

**Files in this Data Supplement:**

- Supplementary Data - zip file
