# Supplementary figures and images for: Mutations in B4GALNT1 (GM2 synthase) underlie a new disorder of ganglioside biosynthesis
Source: Brain. 2013 Oct 6;136(12):3618–24. doi: 10.1093/brain/awt270 (PMC3859217; doi:10.1093/brain/awt270)

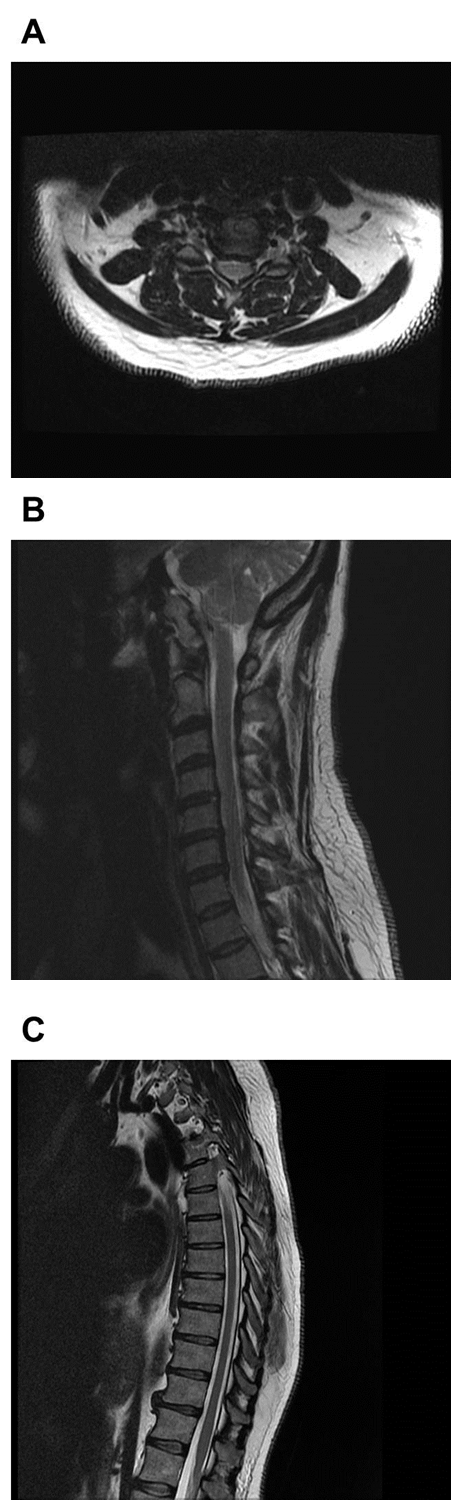

Supplement: Supplementary Data [file supp_awt270_suppl_data.zip › brain-2013-01033-File009.tif]

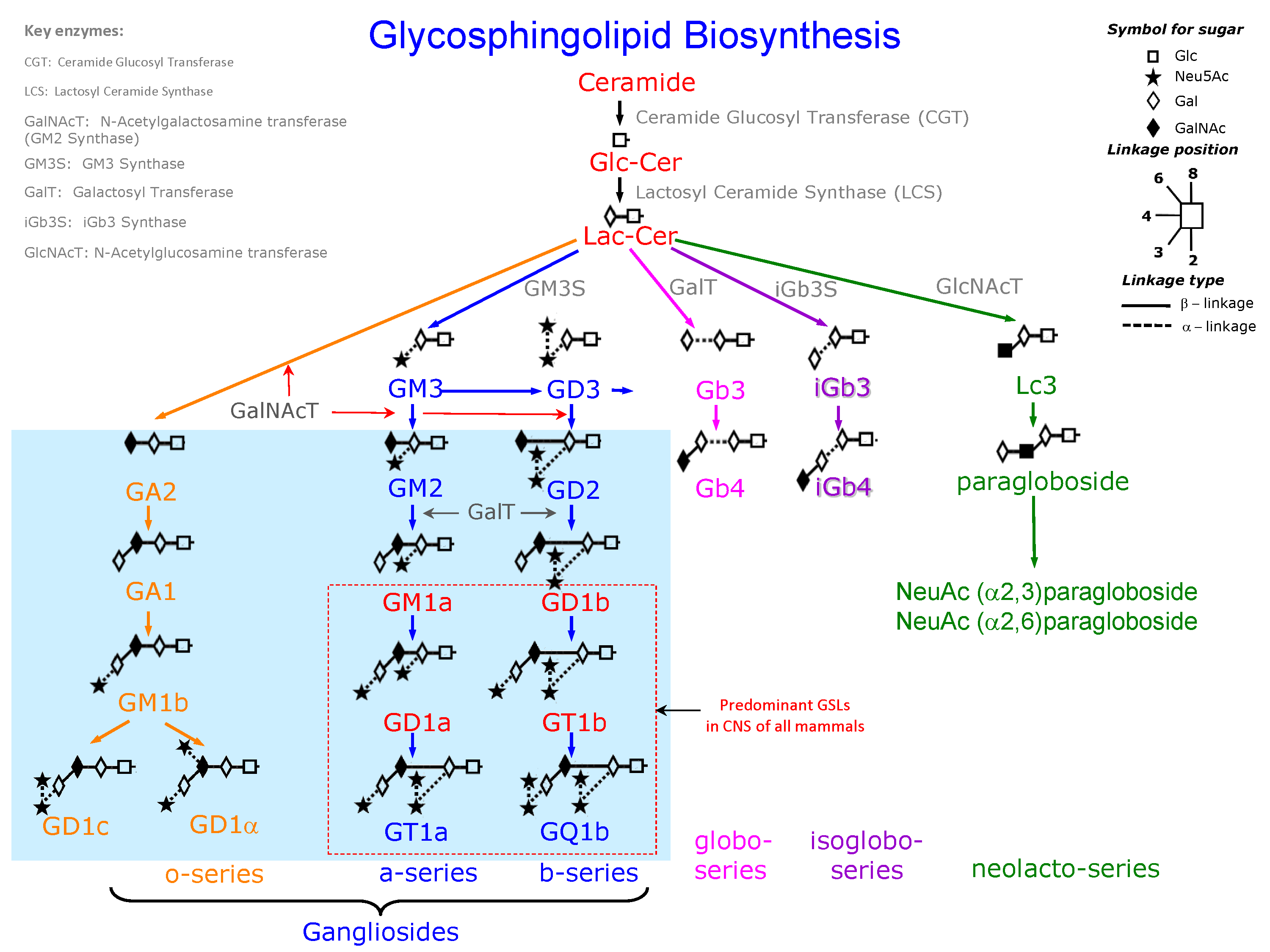

Supplement: Supplementary Data [file supp_awt270_suppl_data.zip › brain-2013-01033-File005.tif]

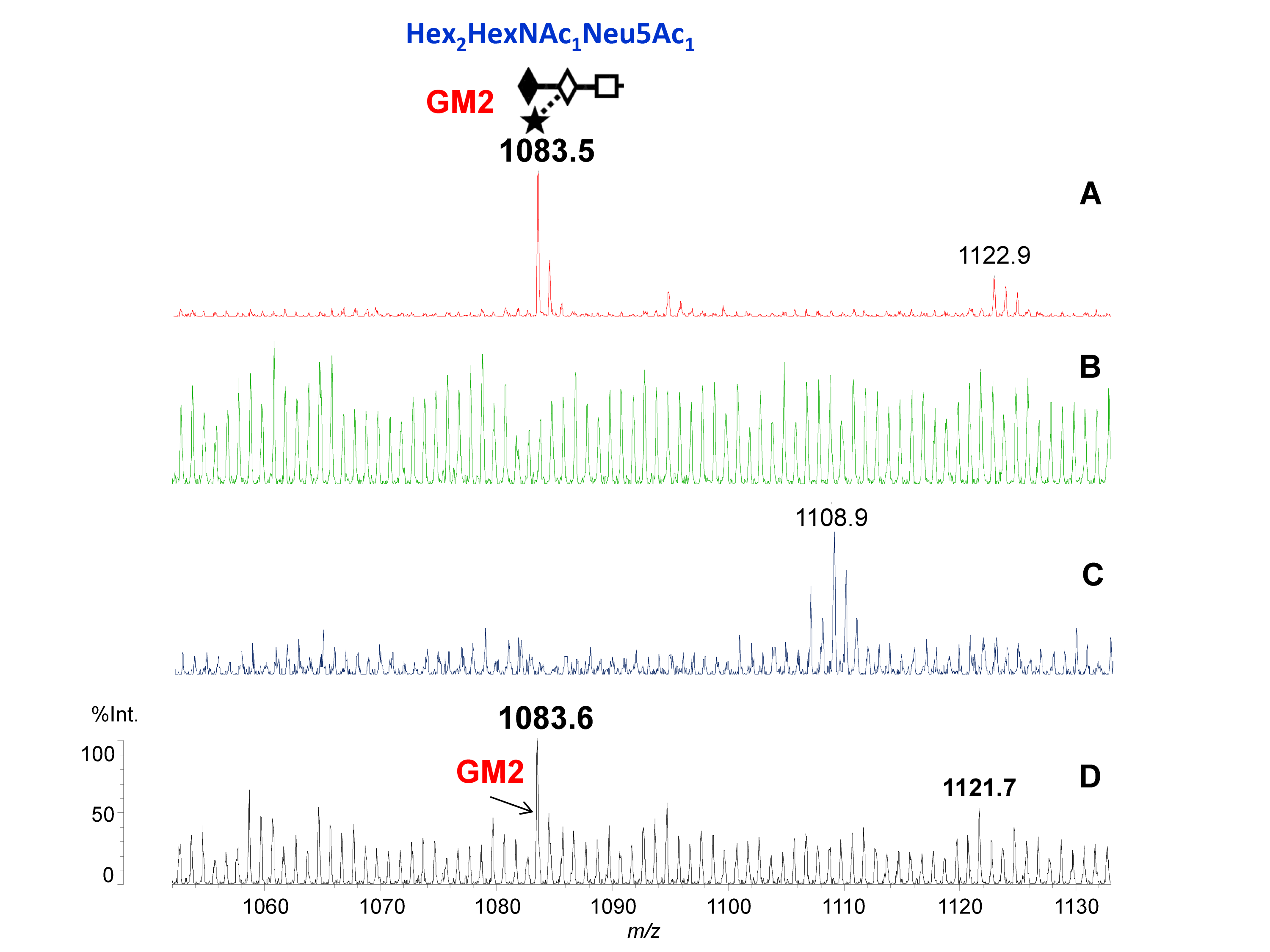

Supplement: Supplementary Data [file supp_awt270_suppl_data.zip › brain-2013-01033-File006.tif]

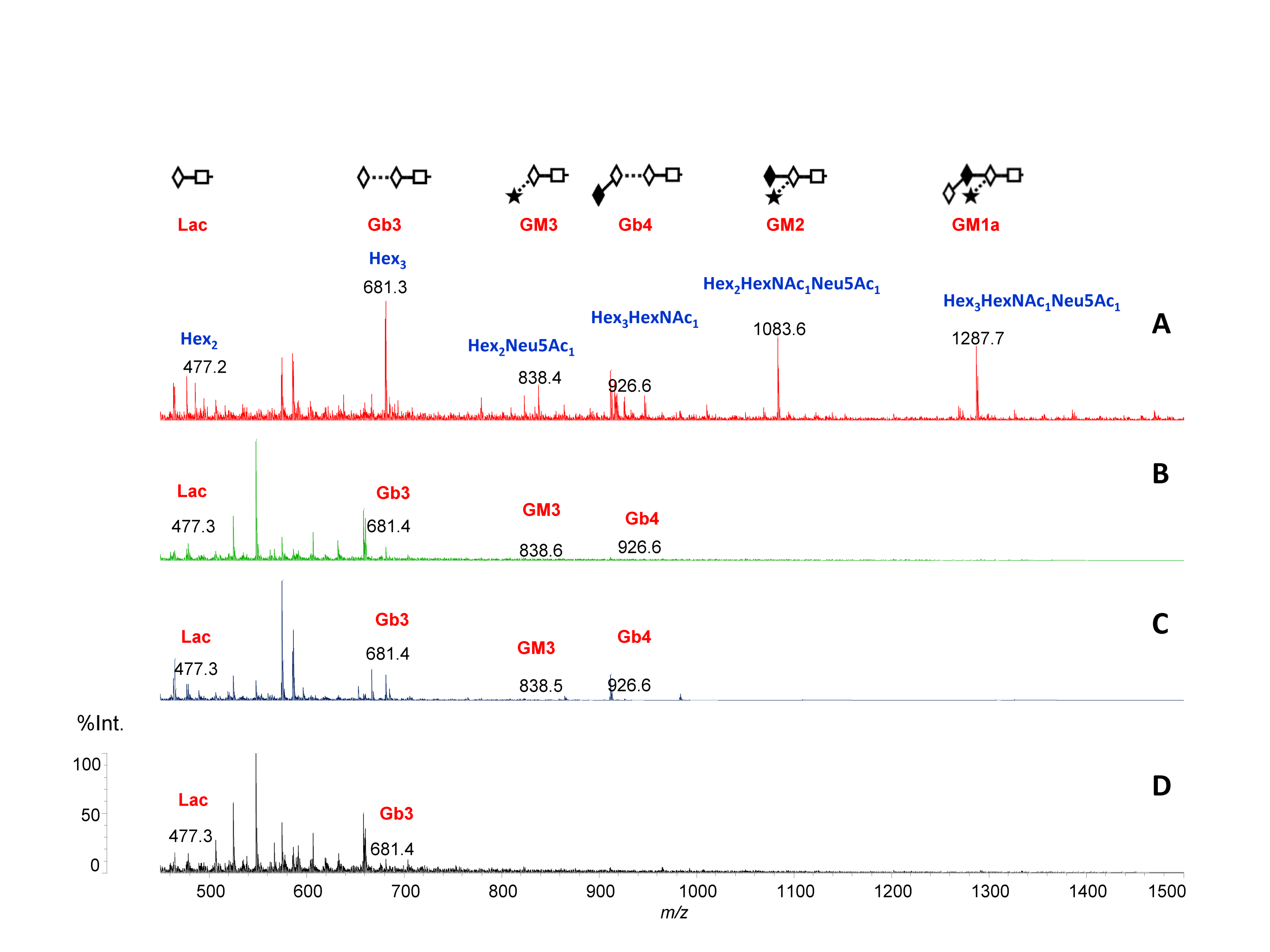

Supplement: Supplementary Data [file supp_awt270_suppl_data.zip › brain-2013-01033-File007.tif]

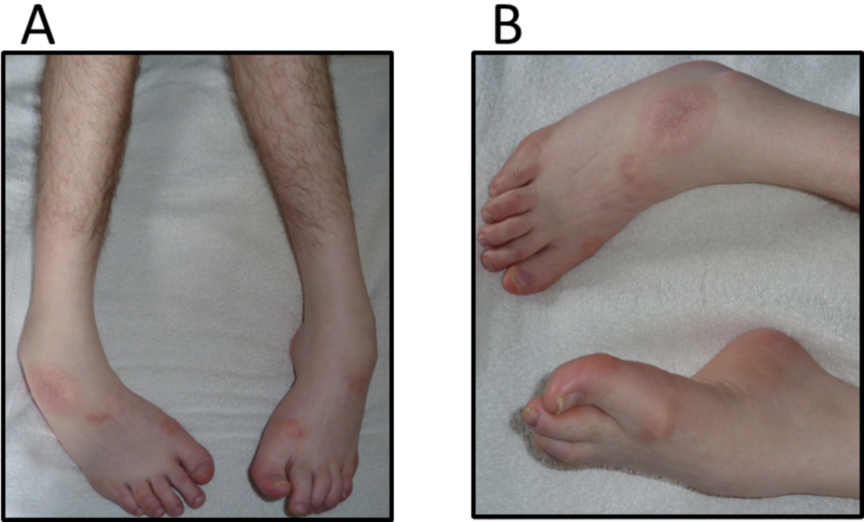

Supplement: Supplementary Data [file supp_awt270_suppl_data.zip › brain-2013-01033-File008.tif]
