# Supplementary material for: Mutations in B4GALNT1 (GM2 synthase) underlie a new disorder of ganglioside biosynthesis
Source: Brain. 2013 Oct 6;136(12):3618–24. doi: 10.1093/brain/awt270 (PMC3859217; doi:10.1093/brain/awt270)
Supplement: Supplementary Data [file supp_awt270_suppl_data.zip › brain-2013-01033-File004.doc]

**Supplementary material**

**Mutations in *B4GALNT1* (*GM2 Synthase*) underlie a new disorder of ganglioside biosynthesis**

Gaurav V. Harlalka,^1,*^ Anna Lehman,^2,*^ Barry Chioza,^1,*^ Emma L. Baple,^1,*^ Reza Maroofian,^1^ Harold E. Cross,^3^ Ajith Sreekantan-Nair,^1^ David A. Priestman,^4^ Saeed Al-Turki,^5^ Meriel E. McEntagart,^6^ Christos Proukakis,^7^ Louise Royle,^8^ Radoslaw P. Kozak,^8^ Laila Bastaki,^9^ Michael Patton,^6^ Karin Wagner,^10^ Roselyn Coblentz,^10^ Joy Price,^6^ Michelle Mezei,^11^ Kamilla Schlade-Bartusiak,^2^ Frances M. Platt,^4^ Matthew E. Hurles^5^ and Andrew H. Crosby^1^

1 Institute of Biomedical and Clinical Science, University of Exeter Medical School,

St. Luke's Campus, Heavitree Road, EX1 2LU, Exeter, Devon, UK

2 Child and Family Research Institute, The University of British Columbia, 950 West

28th Ave, Vancouver, BC, V5Z 4H4, Canada

3 Department of Ophthalmology and Vision Science, University of Arizona School of

Medicine, Tucson, AZ 85711, Arizona, USA

4 Department of Pharmacology, University of Oxford, Oxford, OX1 3QT, UK

5 Wellcome Trust Sanger Institute, Wellcome Trust Genome Campus, Hinxton,

Cambridge, CB10 1SA, UK

6 Medical Genetics Unit, Floor 0, Jenner Wing, St. George's University of London,

Cranmer Terrace, London SW17 0RE, UK

7 Department of Clinical Neuroscience, Institute of Neurology, University College

London, Queen Square, London WC1N 3BG, UK

8 Ludger Ltd., Culham Science Centre, Oxfordshire, OX14 3EB, UK

9 Kuwait Medical Genetics Centre, Ghanima Alghanim Bldg; Maternity Hospital,

Sulaibikhat, Postal Code: 80901, Kuwait

10 Windows of Hope Genetic Information Center, Holmes County, Walnut Creek,

**OH 44065** Ohio, USA

11 Division of Neurology, The University of British Columbia, UBC Hospital S192 –

2211 Wesbrook Mall, V6T 2B5, Canada

*These authors contributed equally to this work.

**Correspondence to:**

**Genetic studies**

Prof. Andrew H. Crosby

Institute of Biomedical and Clinical Science

University of Exeter Medical School

St. Luke's Campus, Heavitree Road,

EX1 2LU, Exeter, Devon, UK

Email: [A.H.Crosby@exeter.ac.uk](mailto:acrosby@sgul.ac.uk)

**Biochemical analyses**

Prof. Frances Platt

Department of Pharmacology,

University of Oxford, Oxford,

OX1 3QT, UK

Email: [frances.platt@pharm.ox.ac.uk](mailto:frances.platt@pharm.ox.ac.uk)

**Running title:** New disorder of ganglioside biosynthesis

**Supplemental inventory**

**1. Supplementary Figures and Tables**

Figure S1

Figure S2

Figure S3

Figure S4

Figure S5

Table T1

Table T2

**2. Supplementary clinical descriptions**

**3. Supplementary references**

**1. Supplementary Figures and Tables**

**Figure S1.** Glycosphingolipid biosynthesis. The shaded blue box shows the gangliosides that are not synthesized in the absence of B4GALNT1 (GM2 Synthase).

**Figure S2.** Representative MALDI profiles (high mass window 850-2000 Da) for permethylated glycans released from GSLs isolated from cultured human skin fibroblasts. Authentic standards (A); Unaffected patient control (D); Affected sister/sibling (B,C) in the Italian family. M/Z=1085.5 (GM2) absent in afffected sister/sibling, but present in unaffected patient control.

**Figure S3.** Representative MALDI profiles (low mass window 300-850 Da) for permethylated glycans released from GSLs isolated from cultured human skin fibroblasts. Authentic standards (A); Unaffected patient control (D). Affected sister/sibling (B,C) in the Italian family. M/Z=1085.5 (GM2) absent in afffected sister/sibling, but present in unaffected patient control.

**2. Supplementary clinical descriptions**

**Clinical descriptions:**

**Family 1:**

Presented previously (Wilkins*on et a*l., 2005).

**Family 2:**

**IV:3** *-* This man, now age 51 years, reportedly had normal early developmental milestones, but was diagnosed with intellectual disability (IQ 64) in childhood. He began tripping easily in his late 20s and sought neurological assessment for progressive lower limb weakness at age 37 years. He had hypertonicity and decreased power of 4 / 5 in the lower limb muscle groups. Sensation was intact clinically, but nerve conduction studies (NCS) showed decreased sensory nerve amplitudes, and normal motor nerve conduction. The deep tendon reflexes were intact and electromyography was normal, consistent with an upper motor neuron problem. MRI of the spinal cord showed congenital spinal stenosis at C3-7 causing mild flattening of the cord, but not significant enough to cause his lower extremity weakness. Brain structure per MRI was normal. Brain MRS showed a nonspecific low N-acetylaspartate peak. A muscle biopsy from quadriceps showed nonspecific changes with Type II fiber dominance. Muscle weakness then progressed quickly over two years, losing the ability to run, and he began to have spasticity with upgoing Babinski responses. Extensor muscle groups were stronger than the flexors. Repeat NCS showed a sensory polyneuropathy. He also had unilateral carpal tunnel syndrome. He was also noted to have bilateral vestibular hypofunction. Normal investigations included lactate, plasma amino acids, urine organic acids, acylcarnitine profile, vitamin E, urine oligosaccharides, hexosaminidase A, B12, TSH, creatine phosphokinase, calcium, electrolytes, creatinine, liver enzymes, blood counts, karyotype, FMR1 repeat length testing, and transferrin isoelectric focusing. His medical history is otherwise significant for adenocarcinoma of the sigmoid colon (resected successfully at age 40 yrs), mild hypertension, hypothyroidism, and dyslipidemia.

He has had ongoing gradual loss of strength in his lower limbs and required a wheelchair at age 51 years. Strength in the upper limbs is preserved. There has been no cognitive decline. He has normal bowel and bladder function. He has never had a seizure. He has a prominent brow, mild prognathism, and a wide mouth.

**IV:4** *-* This woman, now age 45 years, also had mild intellectual disability (IQ 59) identified shortly after school entry. She always had impaired fine and gross motor skills. Frank spasticity and lower limb weakness was first documented at age 39 years. She is still ambulatory and her upper limbs retain full power. Her course of progressive paraplegia has closely followed her brother’s. There has been no confirmed cognitive decline, but her skills have decreased since the onset of psychiatric illness. She, like her brother, has mild prognathism and a wide mouth. She has had resection of bilateral ovarian mature cystic teratomas. Her investigations included normal cerebrotendinous xanthomatosis screening with plasma cholestanol, hexosaminidase, galactocerebrosidase, arylsulfatase A, oligosaccharides, transferrin isoforms, very long chain fatty acids, phytanic acid, acylcarnitine profile, urine pyrimidines and purines, urine organic acids, and urinary mucopolysaccaridoses screen. Brain MRI / MRS was normal. Spine MRI showed a congenital narrowing of the cervical canal.

**Family 3:**

**VII:1** *–* This woman aged 37 years old, has severe cognitive impairment, stereotypical rocking behavior and is unable to self-care or interact socially with others. She is under the care of a psychiatrist for depression and psychotic features. Gross motor milestones were delayed and she walked alone at 19 months, toe walking frequently from the start. Currently she is ambulatory with assistance but prefers to crawl. She has never developed any speech and has symptoms of pica (frequent ingestion of paper). The onset of lower limb spasticity has been progressive, she has bilateral pes cavus, bilateral extensor plantar response and ankle contractures. Clinical examination was limited due to the subject’s psychiatric state.

**VII:2** *–* This man, aged 56 has mild/moderate intellectual disability and features suggestive of an autistic spectrum disorder including stereotypies and poor social communication skills. His developmental milestones were globally delayed. Progressive lower limb spasticity and weakness started in early childhood and he was ambulatory until 13 years ago, currently mobilizing by crawling on his knees. Aged 52 years he has a history of *grand mal* seizures managed successfully with phenytoin treatment. Surgery was performed for bilateral cataracts 10 years ago.

Clinical findings included: hypertonicity and reduced power of 4 / 5 in the lower limb muscle groups, bilateral wasting of the calf muscles, pes cavus, ankle and knee contractures and extensor plantar responses, ankle jerks were absent. There was mild wasting of the thenar and hypothenar muscles, upper limb findings were otherwise unremarkable. He has normal bladder and bowel function.

**VII:5** *–* This woman, aged 47 has mild intellectual disability and emotional lability. Developmental milestones were globally delayed; walking was abnormal from the onset and gradually declined. Lower limb weakness has been progressive and she has been wheel chair bound for 5 years. She has a previous history of *grand mal* seizures, but is currently seizure free.. Examination showed spastic paraperesis with reduced power 4 / 5 in the lower limb muscle groups, bilateral pes cavus and extensor plantar reflexes. Absent ankle jerks and reduced sensation in the lower limbs were suggestive of a peripheral neuropathy, There was mild wasting of the thenar and hypothenar muscles, upper limb findings were otherwise unremarkable. Cerebellar signs included mild symmetrical dysdisdochokinesia and terminal dysmetria.

**IX:1***-* This 9 year old boy has mild/moderate intellectual disability, hyperactivity, poor concentration and social communication skills. He walked alone at 16 months and his parents report poor coordination, he has no discernible gait abnormality at present. Examination findings of the lower limbs included increased reflexes with normal power, there was y shaped 2-3 skin syndactyly of the toes bilaterally, plantar reflexes were equivocal. There were no significant upper limbs findings.

**Figure S4.** (A and B) Wasting of the calf muscles, pes cavus, and calluses over pressure points in subject VII:2 from family 3.

**.**

**Figure S5:** MRI images taken of subject IV:2 from family 2: A)Axial T2 weighted image at C5-6 B) Sagital T2 weighted image of the entire cervical spine. C) Sagital T2 weighted image of thoracic spine. The radiology report stated: There is a generalized reduction of the AP diameter of the cervical canal due to congenitally short pedicles. Broadbased disc osteophyte complex at C5/6 causes moderate cord deformity, mild broadbased disc protrusion at C4/5 causes moderate indentation one at C6/7 also causes mild cord defomity. No increased cord signal. Thoracic cord showed only mild degenerative osteophyte formations and is developmentally normal.

**3. Supplementary references**

Wilkinson PA, Simpson MA, Bastaki L, Patel H, Reed JA, Kalidas K, et al. A new locus for autosomal recessive complicated hereditary spastic paraplegia (SPG26) maps to chromosome 12p11.1-12q14. J Med Genet. 2005;42(1):80-2.
